# Toward Simple, Predictive Understanding of Protein-Ligand Interactions: Electronic Structure Calculations on Torpedo Californica Acetylcholinesterase Join Forces with the Chemist’s Intuition

**DOI:** 10.1038/s41598-020-65984-0

**Published:** 2020-06-08

**Authors:** Nitai Sylvetsky

**Affiliations:** Department of Organic Chemistry, Weizmann Institute of Science, 7610001 Rehovot, Israel

**Keywords:** Chemistry, Biochemistry, History of chemistry, Supramolecular chemistry, Theoretical chemistry

## Abstract

Contemporary efforts for empirically-unbiased modeling of protein-ligand interactions entail a painful tradeoff – as reliable information on *both* noncovalent binding factors and the dynamic behavior of a protein-ligand complex is often beyond practical limits. We demonstrate that information drawn exclusively from static molecular structures can be used for reproducing and predicting experimentally-measured binding affinities for protein-ligand complexes. In particular, inhibition constants (K_i_) were calculated for seven different competitive inhibitors of Torpedo californica acetylcholinesterase using a multiple-linear-regression-based model. The latter, incorporating five independent variables – drawn from QM cluster, DLPNO-CCSD(T) calculations and LED analyses on the seven complexes, each containing active amino-acid residues found within interacting distance (3.5 Å) from the corresponding ligand – is shown to recover 99.9% of the sum of squares for measured K_i_ values, while having no statistically-significant residual errors. Despite being fitted to a small number of data points, leave-one-out cross-validation statistics suggest that it possesses surprising predictive value (Q^2^_LOO_=0.78, or 0.91 upon removal of a single outlier). This thus challenges ligand-invariant definitions of active sites, such as implied in the lock-key binding theory, as well as in alternatives highlighting shape-complementarity without taking electronic effects into account. Broader implications of the current work are discussed in dedicated appendices.

## Introduction

Protein-ligand (PL) interactions have drawn great amounts of scientific attention throughout the last century (see refs. ^1–4^. for a few recent textbooks and reviews). Aside from being examined for playing crucial roles in a variety of essential biochemical processes, such interactions are often focused on in many drug design studies – revolving around finding inhibitors for proteins such as enzymes and neuroreceptors for the purpose of invoking a desirable biological response^5–8^. Due to such considerations, many researchers from a broad spectrum of scientific disciplines (consisting of computational biologists and biochemists as well as theorists from chemistry and physics) have attempted to provide some general theoretical/computational modeling schemes for predicting biochemically-relevant PL binding events^9–13^.

Various protein-ligand binding theories, which underlie many research efforts in the field, have been proposed. The latter include the infamous “lock-key” model, originally introduced by Fischer^14^. This model has subsequently been corrected by Koshland to account for mutual, structural adaptations in both protein and ligand (“induced fit”) – embracing the notion of a “glove-hand” correspondence^15,16^. While more recent adjustments, taking additional conformational and solvent effects into account, have also been introduced^17–20^, none have seemed to move past the intuitive notion of shape complementarity – which clearly has undeniable didactic and predictive value, and has been implemented in a vast amount of fruitful research attempts (both computational and experimental)^21,22^. That being said, the latter notion does not explicitly account for electronic interactions taking place in PL systems; thus, a rather different notion of complementarity, dedicated to interactions of this kind, will be explored in the present paper.

It has been well-established that PL systems are greatly influenced by noncovalent interactions (NCIs)^23–28^. The latter, resulting from subtle electronic effects, are very small in magnitude and cannot virtually be measured by experimental means. Thus, *ab initio* electronic structure methods constitute a precious (and almost exclusive) source of information on biochemically-relevant NCIs – which, in turn, is often used for the parametrization and calibration of more approximate computational modeling techniques (such as DFT functionals and molecular mechanics force fields)^29–33^. In order to avoid empirical biases, one could ideally use such nonempirical electronic structure methods for running molecular dynamics (MD) simulations on realistic PL systems; in such scenario, information drawn from such simulations would include an adequate description of biochemically-significant NCIs, and it can thus be expected to offer desirable predictive power (which is, after all, the main goal of any theoretical model). However, electronic structure calculations are notorious for their steep computational cost scaling with the system’s size (see associated discussion in, e.g., ref. ^34^) – which generally precludes using them for MD simulations on realistically-sized biochemical systems (excluding a few recent approximate approaches, each entailing different methodological challenges; see, for instance, refs. ^35,36^). Thus, molecular mechanics^37–39^ and docking approaches^40–42^ are employed in most practical drug design studies. Such approaches are, for the most part, parametrized based on either empirical data or on results from quantum chemical calculations, and are shown to account for NCIs in an approximate, yet often qualitatively-inaccurate manner – in addition to being prone to errors resulting from training biases^43,44^.

For this reason, and since *some* description of NCIs relevant for PL binding is clearly crucial for predictive purposes^45,46^, electronic structure calculations are usually combined with additional computational techniques used for describing the dynamic, continuous relationship between PL pairs that leads to biochemically-significant (active-site) binding. In this manner, electronic structure calculations are performed on *static* structures, which are assumed to represent crucial events in the PL binding process (see ref. ^47^ for a recent, comprehensive review). It is generally assumed, for instance, that the actual biochemically-significant binding event – taking place in the protein’s active site – must incorporate some description of noncovalent binding factors. Thus, one common piece of information on PL interactions provided by electronic structure methods corresponds to the PL binding energy – calculated as the energetic difference between the bound PL structure and its underlying protein and ligand structures found at infinite separation (Eq. 1):

1 $$\Delta {E}_{bind}={E}_{PL}-({E}_{P}+{E}_{L})$$

**Figure 1:**
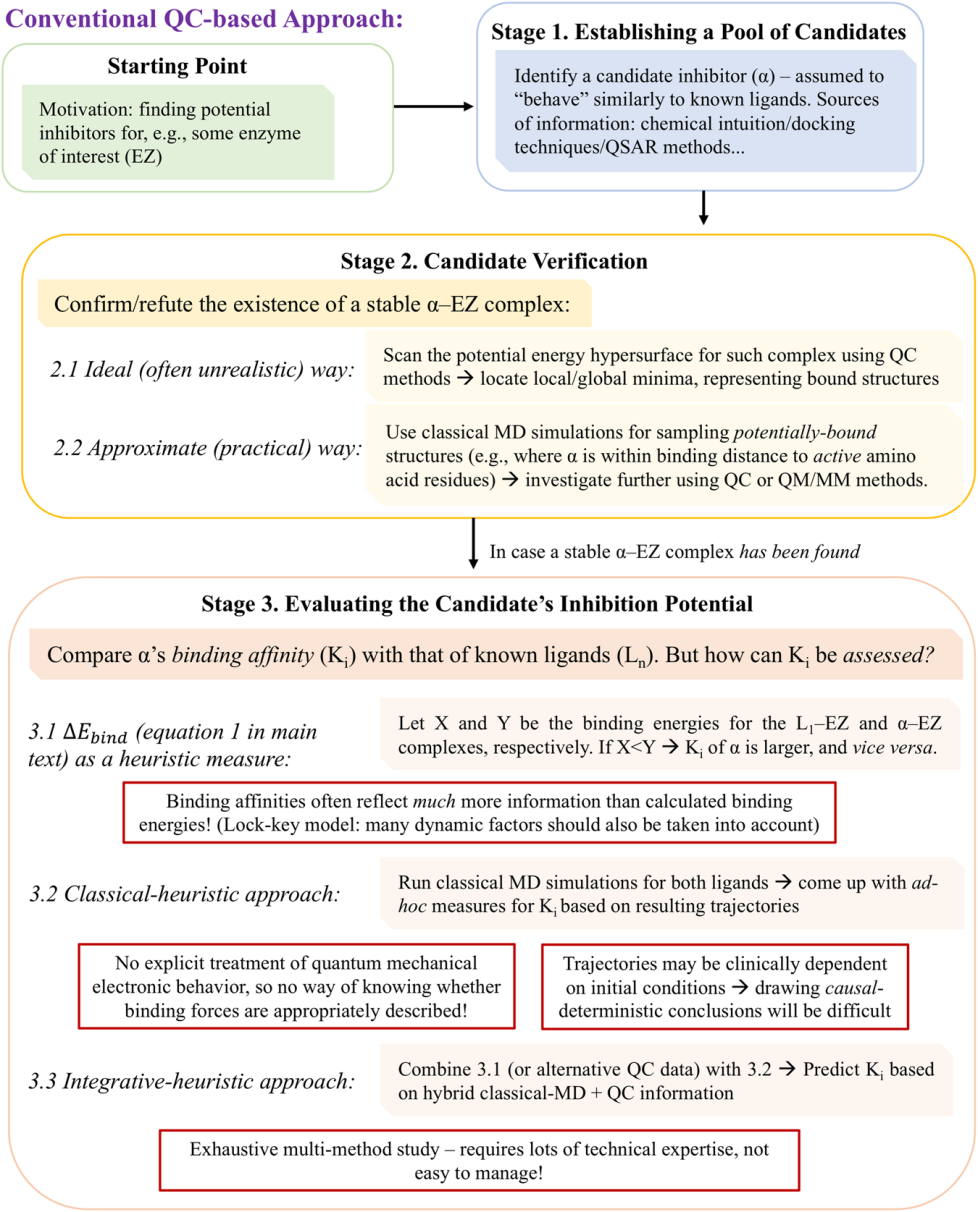
A hypothetical, “conventional” molecular-modeling-based ligand identification process, employing quantum chemistry methods. Compare with Figure 3, which illustrates our approach as proposed in the present paper (Acronyms: QC = quantum chemistry, MD = molecular dynamics, QM/MM = hybrid quantum chemistry – classical molecular mechanics methods. Some crucial problems threatening the process’ success are outlined in red).

Where P and L stand for protein and ligand, respectively (in their complex-structure geometry). It should be pointed out that the relationship between such calculated energetics and realistic PL systems is quite unclear (as said, PL binding is a continuous, dynamic process; representing it using such “binary” means – i.e., bound complex vs. free structures – clearly ignores this fact); still, quite a few authors have employed such quantities as bits-and-pieces of information in more-general predictive theoretical/computational schemes – where additional such pieces, obtained using different techniques (e.g., classical MD trajectories), are also used^48–53^. Needless to say, such multi-method efforts require an appropriate multi-method-expertise from the researcher, and entail lots of (perhaps undetectable) sources of error and technical difficulties – as demonstrated in Figure 1.

Thus, when interested in predictive modeling of PL systems, we are often faced with a painful dilemma: An appropriate description on biochemically-relevant NCIs is, on the one hand, required; the dynamic relationship between PL pairs cannot, on the other hand, be ignored; holding on to one source of information and letting go of the other would make our inquiry simple and elegant, but often wrong and unreliable; trying to hold on to both complicates things further, as reasonable interfaces between different kinds of information must be established – giving rise to many corresponding sources of error that cannot necessarily be assessed.

The main aim of this paper is introducing a path toward solving this dilemma – employing electronic structure calculations on *static* molecular structures that *also provide some important information on the dynamic nature of PL binding processes*. In such manner, it should be possible to avoid using MD simulations altogether and still establish valuable predictive models – which may guide future experiments and drug discovery studies. Being mainly interested in utilizing the information offered by electronic-structure methods, and not in specific state-of the-art data analysis and modeling techniques, we will limit our discussion to a very simple predictive model type – based solely on multiple linear regression (MLR). The latter, incorporating independent variables drawn from *ab initio* electronic structure calculations, will be used for calculating experimentally-measured inhibition constants, or K_i_ values (which are ubiquitously used as a practical measure for binding affinities, and compared across different competitive inhibitors as a relative, realistic biochemical reactivity potential with respect to a specific target protein)^54–59^.

Our assumptions, in this context, may be summarized as follows:

- Noncovalent binding in the protein’s active site corresponds to a critical event in the overall, continuous interaction between protein and ligand pairs; that is, a biochemically-significant (i.e., experimentally-measurable) response cannot occur in the absence of such event.
- A combination of independent energetic components derived from a sufficiently-accurate description (which accounts for noncovalent binding factors) of this binding event is characteristic to a given ligand’s isomeric structure and chemical composition. That is, a significant change in the latter would result in qualitatively-different such components.
- Individual local-energy-decomposition (LED; see Methods and Protocols section) contributions exhibit well-defined intermolecular distance dependence^60^; they therefore incorporate some dynamic information on NCIs taking place in the active site. (Indeed, the latter NCIs result, *inter alia*, from the ligand’s electronic properties; thus, they may also reflect additional, potential PL NCIs – taking place *before* active site binding.)
- For quality-control purposes, calculated quantities should *not* implicitly include information from molecular structures or events that are (even slightly) orthogonal to active-site binding. (interaction energies, which employ *optimized* structures for each of the interacting monomers in *vacuum*, do include such implicit information – as opposed to the inter-fragment binding energies used below).

It should be stressed that the very fundamental principles on which our model lie may simply be traced back to *chemical intuition* – as so many predictive tools, incorporating static molecular structures as a source of information, are still extensively used by the general chemistry community for the purpose of studying realistic, dynamic molecular systems. It may seem, in fact, that explaining *dynamic* processes by means of *static* molecular structures is a general feature that defines chemistry as a scientific discipline. The interested reader may browse through an account of this very notion, as well as of representative chemical explanations in which it is rooted in *Appendix A: Static Solutions to Dynamic Problems*.

It should also be emphasized that in the present paper – which is dedicated to a theoretical-methodological a *proof-of-concept* rather than to the development of statistically-robust protocols for practical drug design research attempts – all geometries for the bound PL complexes under consideration were extracted directly from crystal structures (see Methods and Protocols section below). Indeed, the vast majority of practical drug design studies do not make use of such structures – as they are likely to be unavailable at the time of initial candidate verification/screening. Still, our conclusions should, in principle, be extendable to cases where such structures are derived from reliable geometry optimizations – which are extensively explored and discussed in current literature^61–63^.

## Methods and Protocols

All geometries used in this work were obtained in the following manner:

1. Eight crystal structures of Torpedo californica acetylcholinesterase (Tc AChE), each containing a different bound ligand (a.k.a inhibitor) in its active site, were drawn from the PDB website (see corresponding research papers in refs. ^54–59^).
2. Active amino acid residues, defined to be found within 3.5 Å from any atom in the ligand structure (thus being capable of significantly-interacting with the latter; see, for instance, section 2.2 in ref. ^64^) were selected *via* ‘CONTACT’ analyses included in the CCP4 suite^65^.
3. Residues found in the preceding stage for each crystal structure were then simply taken alongside the corresponding bound ligand to create the final active-site + ligand geometries used throughout this paper. All other residues were simply omitted from the latter.

Single point electronic structure calculations were then performed exclusively on the resulting geometries (which had not been optimized further using additional computational protocols) and thus correspond to “QM cluster” calculations according to the taxonomy used in ref. ^47^. All active site structures are described in Table 1, where active amino acid residues are ascribed to each of them based on the selection process outlined above. It can clearly be seen that different residues are present within interacting distance (3.5 Å) from each of the bound ligands considered, such that no two ligands share an identical active site composition. Thus, it is reasonable to argue that the dataset under considerations is composed of systems reflecting diverse and non-uniform noncovalent binding character. Indeed, such active site definition might seem unintuitive to readers used to ligand-invariant such definitions – being mostly founded on the notion of shape-complementarity as implemented in classical molecular dynamics and docking approaches. However, and as demonstrated in the below sections, such ligand-invariant definitions are not required for the predictive purposes considered in this paper.

**Table 1:** Amino acid composition (Tc AChE numbering) of all active-site structures considered in this paper.

Residue Name	Residue Number	PDB ID/Ligand							
		3ZV7/NHG	1W6R/GNT	5NAU/DZ0	1U65/CP0	5NAP/DZ7	1H23/E12	1H22/E10	1E66/HUX
TYR	70				+	+		+	
GLN	74				+				
TRP	84	+			+		+	+	+
GLY	117						+	+	
GLY	118		+				+	+	+
TYR	121		+		+		+	+	
TYR	130						+	+	
GLU	199		+	+		+	+	+	+
SER	200	+	+						
TRP	279			+	+			+	
LEU	282				+				
PHE	284				+				
ASP	285				+				
SER	286				+				
ILE	287						+	+	
PHE	288		+				+	+	
PHE	290		+						
PHE	330				+		+		+
PHE	331		+	+			+	+	
TYR	334				+				
TRP	432								+
MET	436								+
HIS	440	+	+						+

It can be inferred that the resulting dataset consists of diverse noncovalent binding situations.

As mentioned in refs. ^54–59^, all K_i_ values used in our work were experimentally measured in standard laboratory conditions (22–25 °C, pH = 7.0–7.4). The only exception is for the GNT ligand (PDB ID: 1W6R), for which K_i_ was measured in pH = 8.0. As will be shown in the next section, this particular data point is indeed incompatible with its counterparts and was thus omitted from the MLR models considered below.

All electronic-structure-based energetics considered in this paper were obtained using DLPNO-CCSD(T) calculations and subsequent LED analyses included in the ORCA 4.2 program package^60,66^. The choice of this level of theory is based upon its performance in recent benchmark studies on noncovalent systems^67,68^, as well as on practical considerations and limitations (software licenses currently available to us). “NormalPNO” settings, as well as the def2-SVP basis set^69^, were used for in all calculations. Thus, all data were drawn from LED outputs in the following manner:

- DLPNO-CCSD(T)/SVP inter-fragment binding energies were drawn from the “Sum of INTER-fragment total energies” entry, found in the “INTER- vs INTRA-FRAGMENT TOTAL ENERGIES (Eh)” section in the LED outputs. As a sanity check, we verified that binding energies derived from subtracting the sum of “Intra-fragment total energies” from the “total energy” for a given PL complex (both found in the same section in LED output) produce identical energetic values – as shown in the ESI. Note that different definitions for “binding energies” can be found in the literature (some actually correspond to the “interaction energies” mentioned above); in our case, the term simply corresponds to the difference in total energies between the super-system and its underlying protein and ligand fragments [which satisfies assumption (d) in the introduction].
- Energetic contributions corresponding to LED components arising from electrostatics, exchange and dispersion were extracted from the “FINAL SUMMARY DLPNO-CCSD ENERGY DECOMPOSITION (Eh)” section in the LED outputs. Charge transfer contributions were drawn from the preceding “DECOMPOSITION OF CCSD STRONG PAIRS INTO DOUBLE EXCITATION TYPES (Eh)”. Note that for our purposes [see assumption (c) in the introduction], we were interested in grouping different energetic contributions according to their intermolecular distance dependence; thus, we chose to consider the *sum* of “Charge Transfer 1 to 2” and “Charge Transfer 2 to 1” as the total charge transfer contribution to the binding energy (denoted by E_ct_). Similarly, our account for dispersion corresponds to the sum of the “Dispersion (strong pairs)” and “Dispersion (weak pairs)” contributions found in the LED output.

Note that whereas the nonempirical DLPNO-CCSD(T) method and LED approach are used for generating the data considered in this paper – other methods (such as those based on a perturbation theory formalism) may generally be used for similar purposes^70–73^. It should also be mentioned that the above basis set and PNO domains may rightfully be considered inadequate for quantitatively-accurate electronic structure calculations (resulting in energetics found within 1 kcal/mol from a reliable reference level) of noncovalent interactions in *vacuum*^68^. That being said, it should be stressed that accurate calculation of NCI energetics should *not* be recognized as one of the main goals of the current paper. Instead, we will focus on using the very basic *information* derived from LED calculations for explanatory and predictive purposes. Such goal, as we shall show below, is independent of extreme quantitative accuracy considerations.

In addition to the above calculations, additional relative and absolute energies for all geometries under consideration were obtained using the UFF molecular mechanics force-field^74^, as implemented in the Gaussian16 program package^75^.

Multiple linear regression was carried out using the “Analysis Toolpak” add-in for Microsoft Excel 2018 (Macintosh version); 95% confidence intervals were consistently employed for all resulting models. For reproduction purposes, all relevant geometries and ORCA input files used for this paper are provided in the ESI, alongside a dedicated spreadsheet containing our raw and calculated data.

We would like to suggest that our methodological choices and considerations may be of particular interest on their own (and not just as means for achieving the main, stated goal of this paper); the interested reader may browse through associated methodological discussions, questions and answers – all provided in *Appendix B: Methodological Meditations*.

## Results and Discussion

As a first test-run, and in order to check the commensurability of our collection of data points considered below – we established a series of eight MLR models, incorporating all calculated data for all PL complexes as explanatory variables. Each of these models was fitted to seven of the eight data points considered in our study, such that their resulting fitting quality could be compared – and inadequate data points could accordingly be detected. As shown in the electronic supporting information (Supplementary spreadsheet; “Initial Validation”), the quality of the fitting becomes unequivocally superior, and nearly ideal, in the case where the GNT ligand is omitted from the dataset – thereby resulting in model M3 (see discussion of regressions statistics below).

Indeed, this finding may be ascribed to the anomalous experimental conditions used to measure the K_i_ value for this particular ligand (see Methods and Protocols above), as well as to excessive, direct interactions between C and O atoms in the associated PL complex that are not represented in the rest of the data points – thereby giving rise to a fitting error. Luckily, and as can be seen in Table 1, the only active residue which is present exclusively in the 1W6R/GNT structure is that of [290: PHE]. Hence, removing this data point from our study is not expected significantly change the noncovalent binding landscape under consideration. It will thus be excluded from the rest of our discussion.

Experimentally-measured inhibition constants [expressed as log(K_i_)], and calculated data employed in MLR models below (i.e., inter-fragment binding energies and corresponding LED contributions, all given in kcal/mol), are provided in Table 2.

**Table 2:** Experimentally-measured log(K_i_) values, and calculated data for seven Tc AChE ligands – obtained at the levels of theory specified in the Methods and Protocols section.

PDB ID	Ligand	Log(K_i_) (refs. ^54–59^)	Binding Energy	E_elstat_	E_exch_	E_ct_	E_disp_
3ZV7	NHG	3.079	81.767	50.900	13.564	8.025	14.784
5NAU	DZ0	1.475	49.015	28.012	8.192	4.639	11.611
1U65	CP0	1.415	201.247	112.476	36.497	17.648	46.225
5NAP	DZ7	1.046	19.606	12.764	2.953	2.989	3.577
1H23	E12	0.653	220.404	138.560	34.812	19.335	41.187
1H22	E10	−0.097	243.558	154.083	38.869	23.252	44.367
1E66	HUX	−0.886	147.440	74.542	28.487	10.764	39.633

All energetic components are in kcal/mol.

**Figure 2:**
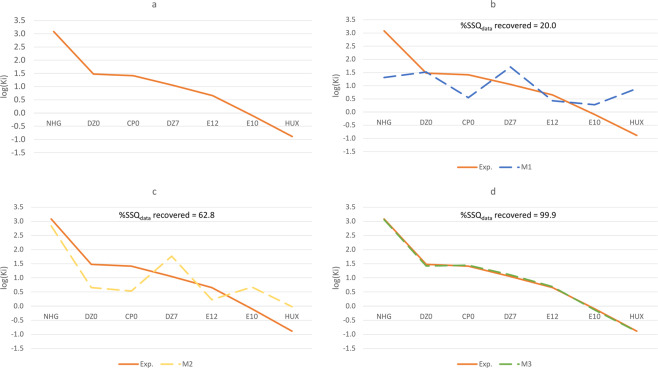
Illustration of regression statistics for the models considered in this work (a) Experimentally-measured K_i_ values (nM) for seven different ligands, taken from refs. ^54–59^. (b) Multiple-linear-regression model [M1] based on inter-fragment binding energies calculated for the above ligands and the corresponding active amino-acid residues in the Tc AChE active site (c) A similar model [M2] based on specific noncovalent interactions calculated for the same systems using the LED approach (d) Our best model [M3], employing both calculated binding energies and specific noncovalent interactions as used in [M1–2]. Clearly, M3 is the most robust model considered – as indicated by statistical parameters (see Table 3) as well as by the similarity between the resulting calculated curve and that of (A).

For illustration purposes, a plot of experimental log(K_i_) values is given in Figure 2a. The explanatory value provided by a simple, MLR-based model M1 – employing calculated binding energies as a single predictive variable – is accordingly demonstrated in Figure 2b. Both residual errors and regression statistics (Table 3) testify that binding energies simply do not possess enough information for reproducing the general trend created by experimentally-measured K_i_ values – as M1 recovers only 20.0% of the sum of squares (SSQ) for the latter. In other words, the variation in K_i_ values is not trivially explained by means of the corresponding binding energies. In addition, residual errors as large as ~1.78 – having clear implications on the model’s explanatory value – can be observed for five of the calculated inhibition constants. These findings clearly fit our expectations regarding the possibility of reducing binding affinities to calculated binding energies – as pointed out in the introduction (see also Figure 1).

**Table 3:** Sum of squares of the data and the residual errors for models M1–3 (left).

	M1	M2	M3	PDB ID	Ligand	eM1	eM2	eM3
**N**_**parameters**_	1	4	5	3ZV7	NHG	1.768	0.241	0.026
**N**_**data**_	7			5NAU	DZ0	−0.045	0.817	0.052
**SSQ**_**data**_	9.59E+00			1U65	CP0	0.866	0.884	−0.029
**SSQ**_**residue**_	7.67E+00	3.56E+00	1.08E-02	5NAP	DZ7	−0.662	−0.723	−0.060
**%Residue**	80.0%	37.2%	0.1%	1H23	E12	0.227	0.424	−0.038
**%SSQ**_**data**_ **recovered**	20.0%	62.8%	99.9%	1H22	E10	−0.376	−0.775	0.036
				1E66	HUX	−1.778	−0.868	0.014
				**MSE**		**1.096**	**0.509**	**0.002**

Particular residual errors (or eM[n], n = 1–3) for the corresponding calculated log(Ki) values are also provided (right).

A similar MLR-based model (M2), based solely on calculated LED components, clearly represents a substantial improvement: it recovers 62.8% of the SSQ for the experimentally-measured K_i_ values (Table 3; Figure 2c). Additionally, residual errors are much smaller compared to M1 – and reach up to ~0.88. The distribution of errors is generally narrower than that of M1 (as also indicated by SSQ_residue_ for each of the models). Such improvements suggest that information representing *particular* NCIs taking place in ligand binding may be used to better explain experimental results – that is, compared to information exclusively drawn from binding energies. Such outcome may partly be attributed to the fact that more informative variables are fitted to approximate the experimental K_i_ curve (the fitting process, however, cannot *exclusively* be held responsible for our models’ explanatory/predictive capabilities, as demonstrated in *Appendix B*). Still, the residual errors and regression statistics clearly preclude this model from being used for practical purposes – as it clearly cannot be used to reproduce the original K_i_ values, neither quantitatively nor qualitatively (ranking ligands based on their calculated binding affinities would deviate from the experimental trend presented in Table 2).

Let us now consider M3, which, as mentioned in the beginning of the current discussion, incorporates *both* DLPNO-CCSD(T) binding energies and LED data employed in M1 and M2, respectively. As shown in Table 3 and Figure 2d, this model clearly exhibits superior performance – as it recovers no-less-than 99.9% of the SSQ for the experimentally-measured K_i_ values. Residual errors are smaller by an order of magnitude, and their distribution is significantly narrower, compared to M1–2: the single-largest error amounts to 0.06, thereby making calculated K_i_ values virtually indistinguishable from their experimentally-measured counterparts. What this means is that the totality of information corresponding to *both* overall binding strength and specific NCI energetics for each of the ligands may be used for reproducing the experimentally-measured K_i_ curve in a satisfactory manner.

In addition to the above discussion, driven mostly by the motivation to emphasize the added explanatory value of LED components to that of total IEs, we executed forward and backward variable selection procedures in order to examine the statistical significance of each of the independent variables under consideration (see Supplementary spreadsheet; “Variable Selection”). We found that excluding any of the five variables incorporated into M3 – all having comparable p values, smaller than $\alpha$ = 0.05 – leads to a rather lethal compromise on accuracy (i.e., a minimum difference of 27% in the %SSQ_data_ recovered by the model, and residual errors as large as 0.9).

Obviously, we do not recommend the above simplistic models for practical predictions of binding affinities – due to the fact that the size and composition of the present dataset cannot possibly allow trivial “extrapolations” to qualitatively-different PL complexes. Nevertheless, we employed a leave-one-out cross validation procedure in order to assess the (external) predictivity of our approach (for a thorough discussion of validation procedures for predictive regression methods, we hereby refer the reader to ref. ^76^). Quite surprisingly, it turns out that even a model as simple as M3, being trained on no more than six data points, exhibits a Q^2^_LOO_ value of 0.78 (see Supplementary spreadsheet; “Cross Validation”). Furthermore, the far-largest prediction error is observed for the HUX ligand – which corresponds to the lowest K_i_ value in the dataset and happens to exhibit rather unique binding characteristics (involving six contacts with five unique amino-acid residues; see Table 1). Prediction statistics greatly improve upon removal of this particular data point (which clearly also leads to a reduction in the total SSQ of the data), leading to Q^2^_LOO_ = 0.91. Thus, since each of the data points corresponds to PL NCIs involving different amino acid residues in the protein’s active site, and despite the fact such cross-validation procedure has its pitfalls compared to more-robust, external validation ones – such result seems to confirm that even a model as simplistic as M3 captures the essential features of the protein-ligand interactions under consideration. It should still, perhaps, be stressed that one should not expect the above straightforward application of our approach to be appropriate for all possible types of PL systems (some particular cases, such as ones involving allosteric effects, are expected to require additional information for establishing predictive capabilities, as discussed in *Appendix B*); we therefore hope to explore more elaborate applications – incorporating additional sources of electronic-structure-based information – in future projects.

The main benefits offered by our above approach may, perhaps, be best illustrated when compared with the corresponding pitfalls associated with molecular-mechanics-based options. As demonstrated in the electronic supporting information (Supplementary spreadsheet; “UFF interaction energies/total energies), alternative MLR models – incorporating either binding energetics or absolute energies obtained using the UFF molecular mechanics force field (which has been parametrized to account for van der Waals interactions) – cannot possibly be used for the purposes considered in the present paper. First, a naked-eye inspection of binding energetics would reveal that NCIs are, in fact, described using a single variable (all energetic contributions except the van der Waals component equal zero); the values the latter takes, however, are clearly less informative than DLPNO-CCSD(T) binding energies – as they can only be fitted to reproduce just 2% (!) of the SSQ for the experimentally-measured K_i_ values. In addition, even an *ad hoc* “kitchen-sink-regression” model, incorporating variables from *total* energy decompositions for the noncovalent complexes under consideration (i.e., to stretching, bending, torsion, out-of-plane and van der Waals components) exhibits poor fitting properties – as it can only be used to cover 53% of this SSQ while making residual errors as large as ~1.4. Thus, and despite containing a larger number of fitted parameters, it is still outperformed by statistically-fragile models such as M2. Needless to say, neither model can possibly be expected to possess any external predictivity value – and should thus be completely disregarded.

At a request of a reviewer, the particular noncovalent forces involved in ligand binding, as described by the aforementioned calculated LED components, will now be discussed. By inspecting the fractions of individual LED components from the corresponding DLPNO-CCSD(T) binding energy (Table 4), it can be seen that despite interacting with different amino acid residues in the protein’s active site – all ligands take part in qualitatively-similar NCIs. First, for all PL complexes considered, it can be seen that E_ct_ $\le$ E_exch_ < E_disp_ < E_elstat_. The relative magnitude of electrostatic contributions ranges between 0.51–0.65 for all systems; it is thus the single, most dominant LED contribution – which can be assumed to dictate the PL binding processes under consideration. The relative magnitude of exchange contributions exhibits very little variation (0.15–0.19), while being slightly smaller than that of dispersion (0.18–0.27). Finally, the share of charge-transfer contributions is the smallest one of all (0.07–0.15). It can therefore be concluded that Tc AChE makes primary use of electrostatic interactions for the binding of all ligands considered above, while exchange and dispersion play additional secondary roles; charge-transfer contributions, however, are lower in magnitude and make the least significant component in the overall PL interaction.

**Table 4:** Relative magnitude of LED contribution x (x = elstat/exch/ct/disp) in the overall binding energy calculated for each of the Tc AChE complexes considered in the present paper (see also Table 2 for absolute values).

PDB ID/Ligand	E_x_/Binding Energy			
	E_elstat_	E_exch_	E_ct_	E_disp_
3ZV7/NHG	0.62	0.17	0.10	0.18
5NAU/DZ0	0.57	0.17	0.09	0.24
1U65/CP0	0.56	0.18	0.09	0.23
5NAP/DZ7	0.65	0.15	0.15	0.18
1H23/E12	0.63	0.16	0.09	0.19
1H22/E10	0.63	0.16	0.10	0.18
1E66/HUX	0.51	0.19	0.07	0.27
**Range**	**0.15**	**0.04**	**0.08**	**0.09**

**Figure 3:**
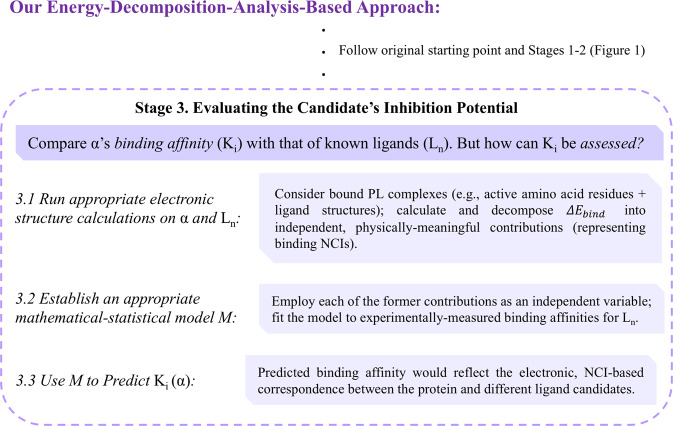
An “alternate ending” to the process presented in Figure 1, making use of our own energy-decomposition-analysis-based approach as outlined above (Acronyms: PL = protein-ligand, NCI = noncovalent interactions).

As discussed in the introduction, the fact that a simple MLR-based model, incorporating information from static molecular structures, can be used to explain/predict complex biochemical phenomena – often said to have infinite degrees of freedom – might seem quite striking. In this context, a few words regarding the scientific *knowledge* gained by the above results should, perhaps, be added. For the sake of the current discussion, let us follow the classic text by Sanders^77^ – which presented knowledge as resulting from the purposeful use of information in an appropriate, well-defined context. Considering the above discussion, a take-home message can be summarized as follows: *static* quantum molecular information may, in principle, be used to provide predictive explanations for *dynamic* protein-ligand processes. This statement clearly has substantial implications on contemporary chemistry knowledge – and we hope it will be of service in future scientific efforts concerning systems of this sort. As mentioned in the introduction, the general idea which underlies our current approach (illustrated in Figure 3) is, by no means, new. Quite a few great chemists have attempted to conduct similar arguments (see *Appendix A*), but seemed to have lacked the appropriate technical means needed for establishing solid, data-based conclusions.

We hereby express our hopes that the basic insight introduced in this paper will, eventually, be implemented in more elaborate and robust modeling techniques – such that desirable external predictivity features will be achieved. As a side note, we would like to mention that our above results, methodological considerations and assumptions may be of interest for several additional reasons (which had not been discussed in preceding sections): [a] physical meaning of LED contributions is different than that of “realistic” NCIs – which do not necessarily exhibit well-defined dependence on the intermolecular distance; in addition, the relationship between such calculated components and the total binding energy is nontrivial; [b] using and validating MLR models for confirming the very *informativeness* of predictive variables is a fundamentally different task than establishing statistically-robust models for practical applications – although the two may easily be confused. Thus, we hereby encourage the reader to browse through *Appendix B*, where such matters are discussed in appropriate length. Finally, and since the derivation of binding affinities from crystal structures is still a matter for ongoing research^78–80^, we would like to propose our approach for such purposes as well. As our above results testify, such desirable goal may indeed be achieved through establishing statistically-robust models – being trained on datasets of appropriate size and later validated to exhibit external predictivity – for the prediction of binding affinities based on calculated NCIs drawn from crystal structured as described in this section.

## Summary and Conclusions

Based on our above investigation of the Tc AChE enzyme and associated ligands, the following conclusions may concisely be summarized:

- We have seen that informative, *static* molecular structures – corresponding to bound protein-ligand complexes – can be used to reproduce the corresponding, experimentally-measured K_i_ values, as well as to predict ones not included in the fitting process. Such findings are, by no means, trivial, since:
  - Binding affinities are assumed to result from a large variety of *dynamic* factors affecting the *continuous* PL binding process.
  - Each of the ligands considered interacts with different residues in the protein’s active site; thus, the resulting performance of a simple multiple-linear-regression model trained merely on several data points suggests that its underlying data should indeed be used for practical predictive purposes.
- Multiple-linear-regression-based models incorporating *either* inter-fragment binding energies *or* LED components calculated for the bound PL structures do *not* possess sufficient explanatory power – as they cover only 20.0% and 62.8% of the sum of squares for the experimental K_i_ values, respectively. In addition, large residual errors (having clear qualitative significance) are observed for both models.
- In contrast, a model employing *both* binding energies and LED components *does* offer desirable explanatory and predictive capabilities, covering no less than 99.9% of the sum of squares for the experimentally-measured values while having negligible residual errors. It also exhibits surprising leave-one-out cross-validation statistics (Q^2^_LOO_=0.78; or 0.91 in case where the HUX ligand, exhibiting unusual binding and statistical characteristics, is omitted), further confirming the practical utility of the explanatory variables considered.
- Active-site structures used in our study – which correspond to amino acid residues found within interacting distance (3.5 Å) from each noncovalently-bound ligand – were shown to possess enough explanatory/predictive power, as demonstrated by the performances of the aforementioned models. This thus challenges ligand-invariant definitions of active sites, such as ones implied in the lock-key binding theory, as well as alternatives highlighting shape-complementarity without taking electronic effects into account.
- When it comes to particular noncovalent forces involved in ligand binding, Tc AChE is shown to make primary use of electrostatic interactions – which amount to a fraction of 0.51–0.65 from the overall binding energy. Exchange and dispersion contributions also play secondary such roles (0.15–0.19 and 0.18–0.27), while charge-transfer contributions are the least significant (0.07–0.15).
- The statistical significance of calculated binding energies and LED components cannot merely be attributed to the number of independent parameters and corresponding fitting coefficients used in each model (*Appendix B, Q2*). Thus, our calculated data clearly has *inherent* explanatory and predictive value.
- Despite the fact that LED components do *not* represent physically-realistic noncovalent interactions (arising from subtle, dynamic electronic effects), they do incorporate highly-valuable information on the latter (*Appendix B, Q1)*. Such information may be combined with additional data (in our case, calculated binding energies) for the purpose of predicting realistic chemical quantities.

Our above conclusions may also be used for adapting the classic “lock-key” analogy to the electronic (noncovalent) PL correspondence examined in this paper: overall active-site binding energetics may be considered to provide some information on a given keyhole’s “size”, while PL complex-specific NCIs (represented by specific LED contributions) incorporate information on its corresponding “shape”. Whereas an entire lock’s mechanism cannot simply be inferred from its keyhole’s properties – focusing on the latter may often suffice for practical predictive purposes. (We hereby remind the reader that analogies of this sort are merely used for facilitating intuitive understanding and should not be taken too literally.)

As a final remark, we would like to express our hopes and great anticipation for additional efforts concentrated on supplying predictive scientific explanations based on chemical intuition (as discussed in *appendix A*). The latter, which may be seen as one of the most prominent achievements of modern science, has apparently not been fully utilized by means of currently-available scientific methods and techniques.

## Supplementary information

Supplementary information1.

Supplementary information2.

Supplementary information3.

## Data Availability

All data generated or analyzed during this study are included in this published article and its Supplementary Information files. In particular, all geometries and ORCA input files used in this work are provided online, alongside a dedicated spreadsheet containing our raw and calculated data.
