# Supplementary material for: Toward Simple, Predictive Understanding of Protein-Ligand Interactions: Electronic Structure Calculations on Torpedo Californica Acetylcholinesterase Join Forces with the Chemist’s Intuition
Source: Sci Rep. 2020 Jun 8;10:9218. doi: 10.1038/s41598-020-65984-0 (PMC7280257; doi:10.1038/s41598-020-65984-0)
Supplement: Supplementary file 1 — Supplementary information1. [file 41598_2020_65984_MOESM1_ESM.pdf]

Toward Simple, Predictive Understanding of Protein-Ligand Interactions: Electronic Structure Calculations on Torpedo Californica Acetylcholinesterase Join Forces with the Chemist's Intuition

Appendices A-B

by Nitai Sylvetsky[*,§]

Email: nitai.sylvetsky@weizmann.ac.il

[§] Department of Organic Chemistry, Weizmann Institute of Science, 76100 Reḥovot, Israel

Appendix A: Static Solutions to Dynamic Problems

The current paper presents an attempt for drawing information on dynamic protein-ligand (PL) binding processes from informative, "static pictures". The latter are assumed to represent critical events, which *must* occur as part of biochemically-significant PL binding (see introduction). It might be important to try and understand why one should, in fact, even expect such "static pictures" to be adequate for describing any dynamic process in the first place.

A realistic chemical sample contains an immense ensemble of molecules, found in various conformations and energetic states. Thus, drawing predictive conclusions from discrete, static molecular structures seems physically-unintuitive. That being said, gathering information describing *all* molecules within the ensemble would be unrealistic. However, what if we could find a way of reducing the behavior of this complicated, realistic ensemble to a few "building blocks", or crucial factors (or pieces of information), represented by a few *specific* static molecular structures? Based on some intuitive assumptions, it should be possible to do so and – in that sense – make large-scale, complicated dynamic phenomena predictively-understandable by relatively simple means.[1,2]

Classical Eyring transition state theory (TST) has, in fact, provided chemists with this very possibility (i.e., reducing dynamic problems to static ones).[3] It allowed, for the first time, the prediction of reaction rates – which reflect dynamic information on "collisions" between molecular species – by means of reduction to static, well-defined molecular structures, corresponding to *critical events* along the reaction path (i.e., the forming of a reactive transition state). Indeed, the theory has been shown to offer great predictive power, despite lacking strict physical rigor in its original form (a fact which did not seem to particularly concern Eyring himself; subsequent, more physically-rigorous versions of the theory are reviewed in Ref.[1]).

The truth is, however, that chemists have been used to solving problems this way long before Eyring has published his theory: it may be argued that predicting chemical properties based on, e.g., relevant Lewis/Kekulé structures is founded on similar logic. Obviously, Kekulé structures of organic molecules cannot be expected to represent how the latter "*look*" in a dynamic environment: the electron density for a given molecule clearly undergoes many changes with respect to the chemical environment, to the point where it is not even trivial to speak of a well-defined molecular structure that is maintained throughout dynamic processes (see, for instance, discussions in Refs.[4,5]). However, *some* static structures *do* interest us

because they provide simple and elegant, practical answers for questions of great importance. Such static structures are therefore of more interest than others, and their identity depends on particular questions we are interested in answering. In the context of chemical reactivity, for instance, energetic *minima* and *maxima* may indeed be of particular interest. Other static structures may allow us to answer questions of different kinds (think, for instance, of solubility; energetic minima/maxima are often less-important than energetically-invariant structural features, such as ones corresponding to an "*ability"* of forming hydrogen bonds). Thus, chemistry may generally be perceived as the science which *reduces dynamic molecular processes to static, informative molecular structures* for the purpose of gaining desirable predictive power (Figure A1). *Chemical intuition* may accordingly be recognized as systematic prediction and rationalization of dynamic molecular phenomena (e.g., chemical reactions, intermolecular interactions, and the like) using static representations of chemical functional units.

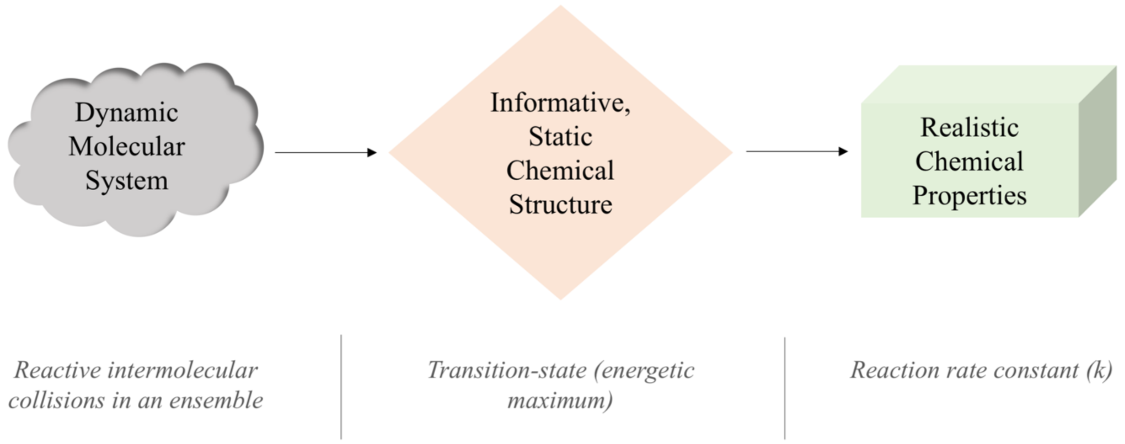

Figure A1. A schematic representation of chemical-intuition-based explanations; specific examples concerning Eyring's transition-state-theory are also provided (below, in *italics*)

Some thoughts regarding harnessing TST to PL systems have been later expressed by Pauling.[6] Trying to rationalize the catalytic "benefit" of enzymatic reactions, he claimed that the latter increase the relative concentration of reactive transition state species compared to corresponding enzyme-free reactions. Regardless of the practical implications for such claim, the motivation behind it may be understood based on the above discussion of TST: instead of bothering ourselves with a vast amount of information associated with the entire PL complex, we may focus on specific features associated with *a single reactive structure*. Thus, if we wish to quantify a given enzyme's catalytic efficiency, all we need to do is compare the relative concentrations of such species in both enzyme/enzyme-free scenarios. Needless to

say, the assumption according to which enzymatic reactions take place *via* single, well-defined reaction paths may indeed be exposed to criticism; however, the same chemical-intuition-driven motivation recognized in the very foundation of TST is again seen to offer great explanatory value. Its practical utility, however, cannot easily be evaluated – due to the fact that finding reactive species of interest using current computational techniques is often impractical.

Since Pauling's above claim had emerged, a few related attempts – based on similar argumentation in different practical "packaging" – have also been published.[2,7–10] Still, searching for reactive transition-state structures, telling the "main story" of continuous PL processes, had continued to be the main bottleneck limiting the latter's applicability. That being said, the fact that practical applications of TST to PL systems have met with many difficulties does not mean that a static representation of such systems, having desirable predictive power, cannot be established. Proving that such possibility exists was, in fact, the main motivation for the present paper. Note that our work corresponds, in a sense, to an *inverse* version of TST – since we use *bound* PL complex structures (i.e., energetic *minima*) as a source of information on dynamic processes. Indeed, it turns out that these structures simply provide enough information for answering practical questions of interest (such as ones requiring comparative assessments of binding affinities). That – from our point of view – plainly stands for yet another useful manifestation of chemical intuition.

Appendix B: Methodological Meditations

In order to facilitate the readers of this text, we hereby provide representative answers for important questions that may seem to arise from it. Both questions and answers have resulted from actual discussions with colleagues from diverse scientific backgrounds.

Q1. LED vs. Physical Realism

- Local-energy-decomposition (LED) components do not represent *realistic* noncovalent interactions (NCIs) – which may not exhibit well-defined intermolecular distance dependencies (*true dispersion*, for instance, cannot be said to have the R^{-6} dependence as E_{disp}; see Ref.[11] and references therein). Besides, considering that no consistent level of theory was used for obtaining all calculated LED contributions – the latter cannot be summed to reproduce the "original" DLPNO-CCSD(T) inter-fragment binding energy values. If that is not enough, geometries drawn from crystal structures do not represent true energetic minima on well-defined potential energy surfaces for the PL complexes at hand. Thus, the data used in models M1-3 consist of approximate quantities that are not deeply rooted in quantum mechanics. How can it then be considered as a reliable source of predictive information?

Indeed, the data used in M1-3 may, perhaps, represent an *approximation* to the "true" NCIs taking place between each ligand and Torpedo californica acetylcholinesterase (Tc AChE). That being said, and putting aside the fact that simple regression statistics confirm the informativeness of our explanatory variables (see Q2 below), it is important to note that we had no intention of restricting our scientific worldview to quantum mechanics. To us, electronic structure calculations are simply a tool for establishing general conclusions – aimed to benefit the science of chemistry. Thus, the use of approximate quantities (which were not derived from a fundamental, consistent physical model – embodied in a single, well-defined electronic structure method) does not harm any of the conclusions drawn in this paper (recall that the main question considered here is: is it possible to reduce PL binding to static chemical structures?).

On a different note, it should be emphasized that despite their seemingly-approximate nature, LED contributions *do* incorporate "high-resolution" information on NCIs – as they are derived exclusively from the nonempirical electronic structure calculated for a given system,

using a well-defined protocol (in this context, see Cioslowski & Surján's "generalized observability criterion" in Ref.[12]). Thus, and despite the fact they are obtained using different levels of theory, the latter may still be calculated and compared *across different systems* for the purpose of arriving at desirable conclusions – as we have chosen to do in this work.

In this context, what really matters is how the above data are being used, and the purposes for which they are used for. Since we have used binding energies and LED contributions as independent variables in multiple-linear-regression-based models, all we care about is comparing their corresponding values across all PL systems under consideration – as opposed to considering different energetic contributions calculated for a single PL complex. For this reason, the strict physical rigor associated with the LED approach is not of our current concern – contrary to its predictive-informative potential. At last, it should actually be mentioned that the fact that the LED contributions do not fully add up to their corresponding binding energies *does represent a practical advantage*; it allows us to avoid intervariable linear-dependence issues that would prevent us from successfully applying multiple-linear-regression (MLR) in the first place.

As previously mentioned in the introduction, all geometries considered in our work simply correspond to informative static structures (*hypothetical* energetic minima, perhaps?), that can be used for predicting experimental binding affinities (that is, to offer *useful and practical understanding* of PL systems). Indeed, finding these structures by means of well-converged, computational geometry optimizations would probably prove to be difficult (for reasons similar to those responsible for the impracticalness of Pauling's idea, as mentioned in *Appendix A*). However, considering the true nature of information and methods used in the present paper, it is possible to conclude that *doing so should not even be necessary*: the crystal structures used by us, which result from statistical averaging of many individual complex structures and clearly do not correspond to such well-defined minima – have provided us with the information we are interested in for achieving our goals. Following a similar logic, previously-unknown informative molecular structures may practically be found through, e.g., semi-converged geometry optimizations combined with intuitive-heuristic principles. The question whether a specific structure truly corresponds to a *physically-realistic* energetic minimum should not also not be of particular concern – as long as it is capable of providing an informative estimate for a specific PL binding energy, as well as for particular NCIs involved in the binding process.

Q2. Causality vs. “Kitchen-Sink Regression”

- In principle, any data may be fitted and used as explanatory variables for reproducing the experimental K_i curve (Figure 2a); so how do you know that your calculated energetics contain information that has some *causal* relationship with the experimental observations?

We have established that binding energies and LED contributions calculated for a set of PL complexes incorporate enough information for reproducing experimentally-measured K_i values in a semi-quantitative manner; what this really means is that the latter may be reduced, *in the statistical sense*, to the former predictors. This does *not* imply, however, that the binding affinity for a given ligand is determined *exclusively* by the physical factors embodied in our calculated data. Nevertheless, MLR is often used to *corroborate* intuitive causal relationships by showing that a given variable(s) incorporates desirable predictive power; for a review of statistical inference and its relationship to causality (which have been thoroughly discussed in many textbooks), we encourage the reader to browse through, e.g., chapter 2 in Ref.[13]

In many practical applications, fitted regression coefficients are interpreted to reflect causal relationships. That is, in cases where residual errors are small enough, and there seems to be an *intuitive meaning* for the particular regression coefficient calculated for each predictor (which depends on the researcher’s expectations) – the relationship between considered predictors and response variable is said to be causal. In the case considered here, coming up with some intuitive meaning for calculated regression coefficients would be rather difficult (see Supplementary spreadsheet; “MLR Models – Ki Values”). That, however, clearly does not mean that our calculated data is uninformative, or that it does not have casual implications on experimentally-measured binding affinities; the contrary, in fact, may easily be confirmed.

In order to quantitatively demonstrate the causal-relevance of our predictors, we compared the predictive-performances of models M1-3 with those of three alternatives (A1-3); the latter have been constructed to incorporate a similar number of independent variables (1,4, and 5, respectively), while the specific values “calculated” for each of the ligands considered are *random numbers* found within the range of corresponding quantities used in M1-3. Thus, the single predictor used in A1 spans randomly-generated values between 20–244 (corresponding to the smallest and largest calculated binding energies used in M1). Similarly, Models A2-3

were established to include random numbers found within the range of the corresponding LED components used in M2-3. It turns out that for a given n (n=1-3), the predictive power of A_n is *substantially lower* than that of M_n (see Supplementary spreadsheet; "A1-3"): A3, for instance, covers just 38.6% of the SSQ for experimentally-measured K_i values (compared to 99.9% for M3). Hence, putting chemical intuition aside – the latter findings may indeed be considered as a fair testimony for the *true informative value* of our calculated energetic properties (which is not related to some statistical-pragmatic notion of causality): it means that the predictive potential of M1-3 cannot be *fully-rationalized* by means of fitting regression coefficients to match a number of observed values.
It should be noted that it *might* just happen that some randomly-generated numbers will prove to reflect predictive information after such fitting process (a fact often incorporated into machine learning techniques[14]) – but that would clearly not teach us anything about the causal relevance of our own calculated data to experimentally-measured K_i values. Needless to say, the DLPNO-CCSD(T)/SVP level of theory used above is, by no means, a random number generator: it offers a well-defined path towards obtaining physically-meaningful energetic quantities. Thus, the fact that the latter provide us with desirable predictive power (which cannot trivially be obtained through optimization of regression coefficients, as done for models A1-3) may also suggest *some* causal relationship between our predictors and the considered PL binding affinities.

<u>Q3. Tc AChE and associated ligands compared to other PL systems</u>

- Some of the reasons for the "push" for dynamic modeling of PL interactions are: (a) when binding of the ligand is dynamically accessible – e.g., the binding pocket is hidden in certain conformations (this may be associated with *allosteric regulation* and other induced conformational changes); (b) the need for including finite temperature effects (such as relevant entropic contributions). To what extent do you think that your approach takes these two factors into account? Would you therefore expect it to be adequate for any possible PL system, or perhaps just for a particular subclass of PL interactions?

First of all, it is worth mentioning that we are *not* claiming that simple models such as M1-3 can be expected to provide useful predictions for just any PL system; while we do believe that our approach (outlined in both main text *and* appendices) should generally be considered

for predictive purposes – we certainly suspect that the particular models considered in this work might be inadequate for some PL cases.
Still, some cases which correspond to reason (a) may also, perhaps, be statistically-reduced to specific NCIs between PL pairs (as binding *specificity* to a particular ligand must incorporate some correspondence of this sort). Similarly, reason (b) would indeed push for dynamic modeling as long as considered effects are expected to qualitatively change the very fundamental PL NCI-based correspondence; if that is not the case – there would be no necessary reason to go beyond the static NCI-based picture. Thus, our approach should, in principle, incorporate the above factors – as long as the PL binding process is dominated by the aforementioned NCI-based correspondence. This would clearly not be true for all possible PL binding processes – as may be illustrated using the following, simple thought-experiment.

Consider, for instance, a protein that introduces some "random" component into the continuous binding process:

- Case #0: Ongoing conformational changes, for instance, may "hide/reveal" the binding pocket independently from any relationship with a particular ligand. Such random component, however, should not – in principle – affect any prediction of *relative* binding affinities (as it is not ligand-specific and should affect any binding process).
- Case #1: Taking an additional step forward, it is possible to think of a protein which introduces some random *and* ligand specific such component. In this case, the bound PL structure may not offer enough information for useful predictions – and additional sources of information would then have to be considered.

We would certainly expect models M1-3 to fail in the latter case. Nevertheless, additional variables (obtained using electronic-structure calculations, or other sources of information) may also be used for arriving at useful predictive capabilities – and so our very general approach should not be suspected for having inherent, system-specific shortcomings.

<u>Q4. Relationship between the current work and QSAR approaches</u>

- Your MLR-based model seems similar to ones used in quantitative-structure-activity-relationships (QSAR) studies. Can you explain how your approach differs from previously-proposed QSAR schemes?

Indeed, it is possible to argue that some QSAR models are based upon a "philosophy" similar to ours; that is, chemical predictions are offered based on calculated structural features that *cannot* be assumed to remain invariant throughout a dynamic molecular process of interest. However, approaches of this sort are, to the best of our knowledge, inherently different than the one used in this work [see assumptions (a)-(d) in the introduction]:[15]

1. QSAR descriptors (explanatory variables) are not often derived *exclusively* from electronic structure calculations on a bound complex structure. Thus, relationships between all predictors and response variable are not rooted in a consistent, uniform or intuitively-understandable physical picture, nor in an actual noncovalent binding event.
2. QSAR descriptors are not explicitly chosen to reflect 'critical events' in particular molecular processes. In fact, particular QSAR descriptors are often chosen based on the statistical robustness of the resulting model alone, while the added information corresponding to these predictors is not explicitly discussed.

The fact that fitting techniques such as MLR are used for constructing predictive QSAR schemes may certainly suggest some similarities between the latter and the simple models (M1-3) used in our work; however, our emphasis lies on electronic-structures- and chemical-intuition-based explanatory variable selection. For all it is worth, it should be possible to say that we have chosen to implement our perception of chemistry in a QSAR envelope. The main idea, however, of drawing predictive information on complex molecular systems from static electronic-structure calculations is independent of such particular implementation and should be considered as the main message of our text.

<u>Q5. Implications on Chemical Bonding</u>

- You attempt to statistically-reduce a continuous, *dynamic* biochemical process to information drawn from selected *static* "electron-density snapshots" of isolated molecular species. It is possible to argue that the very notion of a *chemical bond* is also based on somewhat similar statistical reductions. Should your current effort actually be considered as an attempt for generalizing bonding concepts to PL systems?

Despite not being quite aware of this point in the beginning of our investigations, our above reasoning and inferences do resemble predictive arguments involving notions of chemical bonding. In a sense, chemical bonds do not exist in a dynamic molecular system (as any definition of a "bond" would lie on a static electron-density picture). Still, this concept helps us chemists to arrive at astonishingly-useful practical predictions (of, e.g., reactivity, solubility, and the like – depending on the particular questions considered – as mentioned in *Appendix A*). In the current context, we have chosen to consider ligand "binding" as a "critical (instantaneous, therefore static) event" along a dynamic trajectory, that can be used for predictive purposes; since all we are currently interested in is predicting biochemically-significant responses (i.e., active-site inhibition), "binding" may abstractly be thought of as a set of static pictures which provide us with sufficient predictive power. Such notion of chemical bonding is rather flexible, and may indeed be useful in a variety of (bio)chemical contexts. We surely hope to explore it further in future projects.

Q6. *Ad hominem*

- "I've been in the business for a long while and never witnessed such manuscript getting published. You've got plenty of verbal discussions and no equations to back them up (especially in the appendices). I'm not sure what it is that you're doing, but it doesn't feel like science to me"

Scientific truths do not depend on anyone's feeling of comfort. If you are interested in criticizing the above arguments - please do so while sticking to logic.

References

1. Pechukas, P. Statistical Approximations in Collision Theory. in *Dynamics of Molecular Collisions* 269–322 (Springer US, 1976). doi:10.1007/978-1-4757-0644-4_6.
2. Miller, B. G. & Wolfenden, R. Catalytic Proficiency: The Unusual Case of OMP Decarboxylase. *Annu. Rev. Biochem.* **71**, 847–885 (2002).
3. Eyring, H. The Activated Complex in Chemical Reactions. *J. Chem. Phys.* **3**, 107–115 (1935).

4. Runge, E. & Gross, E. K. U. Density-functional theory for time-dependent systems. *Phys. Rev. Lett.* **52**, 997–1000 (1984).

5. Chattaraj, P. K. & Sengupta, S. Popular electronic structure principles in a dynamical context. *J. Phys. Chem.* **100**, 16126–16130 (1996).

6. Pauling, L. Chemical Achievement and Hope for the Future. *Am. Sci.* **36**, 50–58 (1948).

7. Carter, E. A., Ciccotti, G., Hynes, J. T. & Kapral, R. Constrained reaction coordinate dynamics for the simulation of rare events. *Chem. Phys. Lett.* **156**, 472–477 (1989).

8. Swegat, W., Schlitter, J., Krüger, P. & Wollmer, A. MD simulation of protein-ligand interaction: Formation and dissociation of an insulin-phenol complex. *Biophys. J.* **84**, 1493–1506 (2003).

9. Houk, K. N., Leach, A. G., Kim, S. P. & Zhang, X. Binding Affinities of Host–Guest, Protein–Ligand, and Protein–Transition-State Complexes. *Angew. Chemie Int. Ed.* **42**, 4872–4897 (2003).

10. Tiwary, P., Limongelli, V., Salvalaglio, M. & Parrinello, M. Kinetics of protein–ligand unbinding: Predicting pathways, rates, and rate-limiting steps. *Proc. Natl. Acad. Sci.* **112**, E386–E391 (2015).

11. Giese, T. J. & York, D. M. High-level ab initio methods for calculation of potential energy surfaces of van der Waals complexes. *Int. J. Quantum Chem.* **98**, 388–408 (2004).

12. Cioslowski, J. & Surján, P. R. An observable-based interpretation of electronic wavefunctions: application to "hypervalent" molecules. *J. Mol. Struct. THEOCHEM* **255**, 9–33 (1992).

13. Retherford, R. D. & Choe, M. K. *Statistical Models for Causal Analysis*. *Statistical Models for Causal Analysis* (John Wiley & Sons, Inc., 1993). doi:10.1002/9781118033135.

14. Efron, B. & Hastie, T. *Computer Age Statistical Inference*. *Computer Age Statistical Inference: Algorithms, Evidence, and Data Science* (Cambridge University Press, 2016). doi:10.1017/CBO9781316576533.

15. De Benedetti, P. G. & Fanelli, F. Computational quantum chemistry and adaptive ligand modeling in mechanistic QSAR. *Drug Discov. Today* **15**, 859–866 (2010).
